# Clinical profile and outcome of diabetic foot ulcer, a view from tertiary care hospital in Semarang, Indonesia

**DOI:** 10.1080/2000625X.2017.1312974

**Published:** 2017-05-17

**Authors:** Tjokorda Gde Dalem Pemayun, Ridho M. Naibaho

**Affiliations:** ^a^ Subdivision of Endocrinology, Metabolism and Diabetes, Department of Medicine, Medical Faculty of Diponegoro University, Dr. Kariadi General Hospital, Semarang, Indonesia; ^b^ Resident of Internal Medicine, Medical Faculty of Diponegoro University, Dr. Kariadi General Hospital, Semarang, Indonesia

**Keywords:** Diabetic foot ulcers, clinical profiles, outcomes, Indonesia

## Abstract

**Background**: This study attempted to determine the disease burden in terms of clinical profile and outcome of diabetic foot ulcer (DFU) admissions at a tertiary care hospital in a developing country.

**Methods**: In this descriptive study, the data were collected from the medical record of diabetic patients with foot ulcer who were treated in Dr. Kariadi General Hospital during a 3-year period. The demographic characteristic, type of foot lesion, etiology, isolated microorganism, treatment, and outcome were reviewed.

**Results**: Foot problems accounted for 16.2% of total diabetic admission (*n* = 1429). All patients had type 2 diabetes with no gender predominance. The mean age was 54.3 ± 8.6 years and diabetes control was very poor. Before admission, the ulcers had already developed for 4.7 ± 2.9 weeks; however, the majority of patients were unaware of the preceding causes. Ulcers were neuropathic in 42.2% of cases, neuroischemic in 29.9%, and pure ischemic at lesser percentage. More than 70% of ulcers were in Wagner grade ≥3 with infection event in nearly all patients. The most common isolates from culture were Gram-negative bacteria. A total of 98 (36.3%) lower extremity amputations (LEAs) at various level of the foot were carried out, including major LEA in 24 patients and multiple amputations in seven patients. Mortality rate due to DFU reached 10.7%.

**Conclusions:** Diabetic foot problems constitute a source of morbidity, a reason for LEA surgery as well as being a cause of death among patients with diabetes mellitus.

## Introduction

Diabetes is one of the most prevalent chronic diseases: in 2010, one study reported that 285 million adults worldwide had diabetes and this figure is projected to rise to 439 milion by the year 2030 [1]. Such a profound demographic shift is likely to yield a corresponding increase in the prevalence of diabetes chronic complications, including those in the lower extremity, the diabetic foot [2]. It is estimated that the annual population-based incidence of a diabetic foot ulcer (DFU) ranges from 1.0% to 4.1%. The lifetime incidence may be as high as 25% [3]. Despite the efforts of conservative therapy, there will always be a percentage of ulcers that necessitate hospitalization. These cases may require surgical debridement, resection of distal osseus and soft tissue structure, endovascular intervention, daily dressings, strict glycemic control, and intravenous antibiotic therapy for eradication of infection [4,5].

Foot problems in diabetics can frequently be life or limb threatening, yet have not received the same level of attention as other diabetes complications [6]. Until today, descriptive data regarding demographical and clinical factors in foot ulcers among diabetic patients in Indonesia are relatively few though we are all aware of its clinical importance [7,8]. In the current study, we attempt (1) to record the clinical profile and outcome of diabetic foot hospitalization, and (2) to provide a report which may become a reference for further improvement in diabetic foot management in our center, in Semarang city, Indonesia.

## Methods

### Research design

This study is a retrospective study reviewing the medical records of diabetic patients who were admitted to the Dr. Kariadi General Hospital with foot problems. Dr. Kariadi General Hospital is a provincial hospital, which is also the central referral in Central Java and main teaching hospital of the Medical Faculty of Diponegoro University. Patients with various diabetic complications are referred to this hospital. The study was conducted over a period of 3 years between January 2012 and December 2014 of which the studied population have already been referred to our previous publication [9]. Sampling technique was non-probability with total sampling [10]. Prior to the commencement of the study, ethical clearance was obtained from the hospital authority (number: 269/EC/FK-RSDK/2015).

### Treatment settings

DFU is a general term used to designate lesions that occur in a diabetic patient’s foot, an anatomical area below the malleoli [9]. We hospitalized DFU patients with progressive infection, those with septicemia, gangrenous tissue requiring amputation, need of complex wound care, or when intercurrent disease necessitate it. Endocrinologist or surgeon will take a lead as a physician in charge and treat the patients in close proximity; the strategies were aimed at optimizing the limb salvage rate and healing time by combining first-line medical treatment, revascularization if needed, surgical management, and second-line limited lower extremity amputation (LEA) surgery. Though the largest in-hospital service in Central Java province, our center is not considered a diabetic foot specialty center, which we are currently working on becoming.

On admission, immediate measures were taken for the control of hyperglycemia with insulin. All patients were advised for bed rest or with crutches for pressure relief of ulcers, and casts were only available in few patients who can afford it. Antibiotic was instituted when there were clinical signs of infection. Those with a superficial skin ulcer were treated conservatively by local care (debridement, dressing) while abscess collection was incised and drained. All infected lesions were debrided promptly. Multiple debridement were occasionally required to control infection. Osteomyelitis without peripheral arterial disease (PAD) was treated by limited bone resection. When severe PAD was present, revascularization was done after angiography as soon as patient’s general condition was improved by the medical treatment. Gangrene was treated by amputations at various levels. Life-threatening conditions with severely infected ischemic limbs were also indication for LEA.

### Data collection and procedure

We are greatly helped by the Dr. Kariadi General Hospital’s database to provide the medical records number and 10th International Classification of Disease (ICD-10) diagnosis. A diagnosis of diabetes mellitus was defined as at least one record of ICD-10 code E10 for type 1 and E11 for type 2 diabetes. Diabetic foot problems were confirmed by one of the following physician’s handwritten diagnosis as: ‘diabetic foot’, ‘diabetic foot ulcers’, ‘diabetic foot infections’, or ‘diabetic foot gangrene’. A structured questionnaire was designed and constructed in sections to collect different aspects of the following information: (1) demographic data, (2) diabetes history, (3) laboratory results, (4) diabetic foot investigation for ulcer severity, the presence of infection, peripheral neuropathy and PAD, (5) microbiological profile, (6) treatment, and (7) observed outcomes including the specific levels of LEA, length of stay, and in-hospital mortality. Multiple entries, as detected by their hospital numbers, were treated as separates outcomes as they may have represented new events though occurring in the same patients.

The initial random blood glucose (RBG), fasting plasma glucose (FPG), and glycosylated hemoglobin (HbA1c) following admission were recorded. The results of HbA1c was stratified in percentage graded as per our national guidelines [11]: HbA1c < 7%, good glycemic control; HbA1c 7-7.9%, fair control; HbA1c 8-9.9%, poor control; HbA1c ≥ 10%, very poor control. Specimens of the foot lesions, after decontamination and debridement followed by curettage, were collected for gram stain, aerobic and anaerobic culture, and for antimicrobial susceptibility testing. Radiographs were taken from the affected foot(s) to discover bone abnormalities. Osteomyelitis was determined by radiological examination.

The staging of the ulcers was also reviewed and only the Wagner system [12] was used in this study as this method is the most commonly used in our center. Details of current and past foot disease were recorded, giving attention to the presence of PAD and peripheral neuropathy. PAD was diagnosed as ankle brachial index (ABI) < 0.9 in either leg using a bidirectional handheld Doppler ultrasound instrument (Huntleigh Diagnostics, Cardiff, UK). Peripheral neuropathy was defined as reduced vibration (by 128 Hz tunning fork) or light touch perception evaluated using a 10 g Semmes–Weinstein monofilament (Huntleigh Diagnostics, Cardiff, UK), as previously described elsewhere [9]. Using the clinical information obtained, the type of foot lesion was determined and classified as neuropathic, ischemic, neuroischemic, or unclassified.

### Patient management and outcomes

Patients were evaluated and managed according to Wagner classification, considering the severity of infection and the presence of PAD at the time of presentation. Length of hospital stay was calculated from admission to discharge date. Patient outcomes were classified as:

- conservative management with significant wound improvement (incorporating strict bed rest, intravenous antibiotics, and inpatient podiatric assessment and treatment);
- dressing with or without skin grafting;
- angioplasty or bypass procedure;
- operative procedure: surgical debridement and/or incision drainage followed by wound care, with or without skin grafting; osteotomy; minor LEA: forefoot amputation (digital disarticulation, ray amputation), midfoot amputation (transmetatarsal, Lisfranc’s, Chopart’s), hindfoot amputation (Syme’s); and major LEA: transtibial amputation and transfemoral amputation;
- death in hospital during the episode associated with their diabetic foot complication.

Postoperatively, most patients needed repeated dressing changes and wound toilet. Patients were discharged on the basis of good clinical evidence of wound healing with the elimination of sepsis and necrotic tissue. Post-discharge care ensured adequate off-loading and domiciliary support with regular out-patient clinic attendance.

### Statistical analysis

After the medical records within the predetermined period have been reviewed thoroughly, the collected data was first checked manually for its completeness. Our method was to include all of the available data to describe the population characteristics [13]. Incomplete data were deleted, however, throughout the records were kept to provide an epidemiological information such as demographic data, microbiological study, level of amputation, and mortality rate. Patients with case records reporting a declined amputation (*n* = 9) were not included in the analysis. The data was summarized and then presented in the form of diagrams, tables, and bar charts as appropriate. Data were analyzed using the Statistical Package for Social Science software [IBM version 21.0; SPSS Inc., Chicago, IL, USA].

## Results

There were 232 admissions with diabetic foot problems involving 189 patients; these concerned 96 males and 93 females. Some patients (*n* = 27) were admitted to the hospital more than once for a new lesion. Diabetic foot complications accounted for 16.2% of diabetic admission (*n* = 1429) and 3.0% of the total inpatient population (*n* = 7688) during the 3-year period. The reason for admission included uncontrolled foot infection (69.3%), cardiovascular events (9.3%), hyperglycemic emergencies (5.1%), worsening renal function (3.2%), acute critical limb ischemia (2.8%), hypoglycemic event (2.8%), and others (7.5%). The majority of patients were admitted through the emergency department, while only 11 patients were admitted through outpatient clinic.

### Clinical characteristics of patients

**Figure 1.**
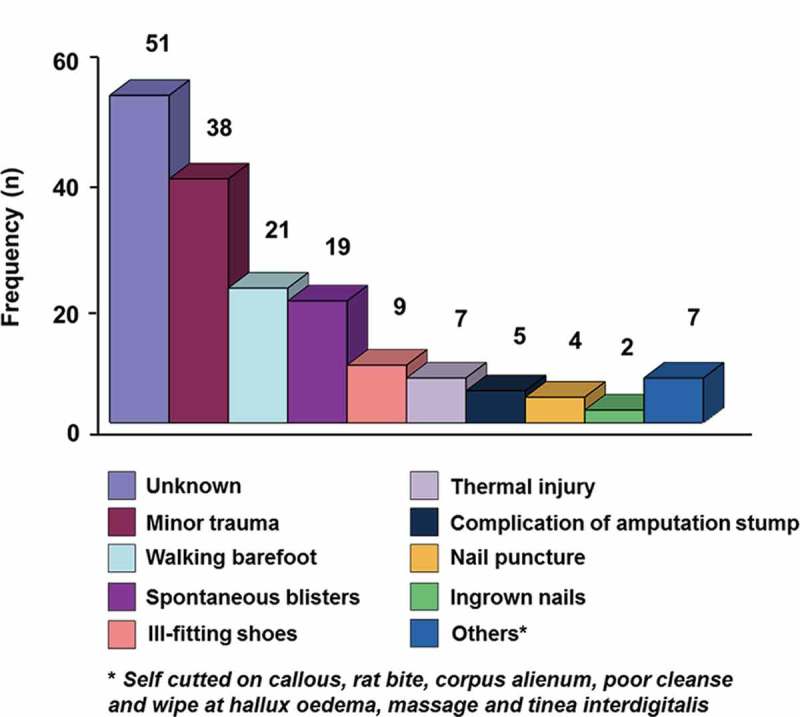
Precipitating events preceding the ulcer.

Table 1 summarizes the demographic and clinical features of patients (*n* = 130). The patients had type 2 diabetes mellitus with the age range between 34 and 79 years. The average duration of diabetes was 6.4 years. None of these patients classified as having type 1 diabetes. The mean HbA1c at the time of admission was 11.3 ± 2.8%. Sixteen patients (12.3%) were newly diagnosed with diabetes. Out of 130 patients, 35.4% of patients developed recurrence of foot ulcers either at the same site or at a different site and had to be hospitalized again. Common precipitating events of ulcers, which are shown in Figure 1, include minor trauma, walking barefoot, spontaneous blisters, and ill-fitting shoes. It was found that 23.7% of patients could not remember the initiating events of the wound. In this study, 154 (71.5%) patients were in high grade Wagner, i.e. Wagner grade ≥ 3 (Table 2). Foot ulcers in 76 patients were pure neuropathic, 19 patients had ischemic-type, while 53 had neuro-ischemic origin. Infection was present invariably in nearly all patients, except four cases with dry gangrene. Among 132 cases with X-ray report of the affected foot, 46 (34.8%) had osteomyelitis. In its most severe form, 13% patients presented with septicemia.

**Table 1.** Demographic details and clinical characteristics of DFU patients, *n* = 130.

	Overall
Age (year)	54.3 ± 8.6
Male /female (*n* = 189)^a^	96/93
Duration of ulcers before admission (week)	4.7 ± 2.9
Type of ulcer (*n* = 177)	
Pure neuropathic, *n* (%)	76 (42.9%)
Pure ischemic, *n* (%)	19 (10.7%)
Neuroischemic, *n* (%)	53 (29.9%)
Non-classified, *n* (%)	29 (16.4%)
History of ulceration, *n* (%)	46 (35.4%)
Previous amputation, *n* (%)	24 (18.5%)
Diabetes duration (year)	6.4 ± 4.9
Newly detected, *n* (%)	16 (12.3%)
<1 year, *n* (%)	5 (3.8%)
1–5 years, *n* (%)	41 (31.5%)
5–10 years, *n* (%)	46 (35.4%)
>10 years, *n* (%)	22 (16.9%)
Admission BG (gr/dL)	359.3 ± 235.9
FPG (gr/dL)	199.2 ± 81.7
HbA1c (%)	11.2 ± 2.8
≤7% (good control), *n* (%)	5 (3.8%)
7.1–8% (fair control), *n* (%)	16 (12.3%)
8.1–10% (poor control), *n* (%)	31 (23.8%)
>10% (very poor control), *n* (%)	78 (60%)
Hospital stay (days)	17.8 ± 10.1
In-hospital mortality, *n* (%)^b^	10.7%

**Abbreviations**: BG, blood glucose; FPG, fasting plasma glucose; HbA1c, glycated hemoglobin.

^a^ Total patients during 3 years period.

^b^ There were 23 mortality rates from total of 215 admission because of DFU.

**Table 2.** Distribution of foot lesion in accordance with Wagner grading system at presentation.

Wagner grade	Signs	*N*	%
0	No ulcer in a high-risk foot	1	0.4
1	Superficial ulcer involving the full skin thickness	5	2.3
2	Deep ulcer penetrating to ligaments/muscle, but no bone involvement or abscess formation	51	23.7
3	Deep ulcer with cellulitis or abscess formation, often with osteomyelitis	71	33.0
4	Localized gangrene	70	32.5
5	Extensive gangrene involving the whole foot	13	6.0
Missing data or not stated		4	1.8
Total		215	100.0

### Bacteriologic pattern

A total of 154 specimens were cultured which yielded 114 positive cultures (74%). Nineteen samples were polymicrobial infection with a total 138 isolates recovered that included 133 bacterial and five fungal organisms. Among the positive-cultured specimen, Gram-negative bacilli constituted the majority (70.8%) and *Escherichia coli* was found to be the most common isolates. Anaerobic microorganisms constituted to 4.3% of the total isolates. The most commonly isolated anaerobe was *Peptostreptococcus spp*.; however, the exact proportion of anaerobes microorganisms may be an underreporting.

### Surgical management and lower extremity amputation

**Figure 2.**
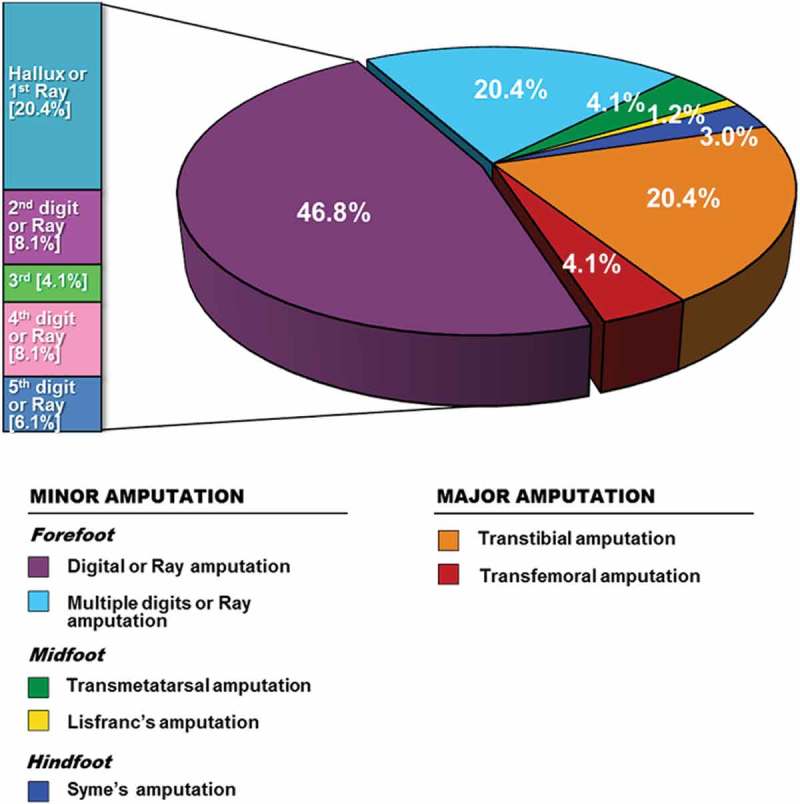
Specific level of lower extremity amputation in diabetes, *n* = 98.

Management strategies for the patients stratified by Wagner classification are shown in Table 3. The most common surgical procedures were debridement and abscess incision and drainage as majority of ulcers were in Wagner grade 3 and grade 4. Patients with only soft tissue wounds (6.9%) were treated by means of local wound care and off-loading measures without requirement of a trip to the operating room. Arterial bypass surgery, specifically femoro-popliteal bypass, was performed in two cases. Primary angioplasty was performed in seven cases (<10% of patients with PAD) with almost half requiring removal of one or more digits. The overall amputation rate was 36.3% (*n* = 98) and specific level of procedures are depicted in Figure 2. Multiple LEA (patients requiring further amputation due to progressions of the disease, such an initial digital and later requiring trans-metatarsal or major amputation) were performed in seven patients. Minor LEA was performed in 74 cases, wherein 67.2% of all amputations were performed at toe level. Major LEA was the outcome for 24 patients with four (4.1%) undergoing above-knee and 20 (20.4%) below-knee amputation.

**Table 3.** Surgical intervention by Wagner grade.^a^

	Wagner grading, *n* (%)						
Type of surgical intervention	0	1	2	3	4	5	Total
Conservative management, *n* = 117							
Daily wound care	0	4	8	3	0	0	15
Debridement	0	5	50	44	2	0	102
Arthrodesis	1	0	0	0	0	0	1
Skin grafting	0	3	5	0	0	0	8
Incision/drainage	0	0	1	31	7	0	39
Osteotomy	0	0	0	10	0	0	10
Revascularization + stenting	0	0	2	4	1	0	7
Arterial bypass surgery	0	0	0	2	0	0	2
Lower extremity amputation, *n* = 98							
Minor LEA	0	0	1	22	51	0	74
Major LEA	0	0	0	2	9	13	24
Total LEA	0	0	1	24	60	13	98

^a^Please note that some patients had multiple procedures in one course of hospitalization, so the frequency is not presented as percentage.

### Management outcomes

The mean length of stay per admission for foot problem was 17.8 days (range from 5 to 71 days). There were 23 deaths among the study sample making the mortality rate of 10.7% and sepsis was the main cause (59%). Those who expired, 10 patients had Wagner grade 3, 11 patients with grade 4, and the remaining are in grade 5 ulcer. Surgical intervention was made only in 14 patients and was not possible in the rest due to their general condition being unfit for surgery. In this report, 13 patients (6.0%) took discharge against medical advice due to variable reasons such as financial constrain, undue delays while awaiting surgical procedure, and refusal of treatment offered, including amputation.

## Discussion

Abbott et al. [14] reported that more than 2% of diabetic patients will develop new foot ulcers annually. The prevalence of DFU varied between 4% and 20.4% among hospital-based studies in individuals with diabetes [15,16]. According to some authorities [17,18], diabetic foot problems are responsible for 23–50% of the hospital bed occupancies by diabetic patients. Our study documented a 16.2% prevalence rate of DFU among consecutive, unselected diabetic patients admitted to the largest medical inpatients service in Semarang, Indonesia. These patients have a significant risk of poor-healing ulcers, foot infection, and LEA, which is reportedly more frequent among low socioeconomic group patients with precarious hygiene conditions [19].

In our study, the proportions of both sexes were relatively comparable as well as middle-aged (mean age 54.3 years). Due to lack of education on nature of illness, they presented to the hospital after 4 weeks after the ulcers had developed. In a study by Lavery et al. [20], duration of ulcers > 30 days was a factor related to development of a wound infection. In our report, infection was present invariably in nearly all patients and Gram-negative bacteria were the most commonly isolated. *Peptostreptococcus spp*. was the most predominant anaerobic isolates, which is in accordance to the previous bacteriologic study from Singapore [21] or other tertiary care hospital in India [22].

Boyko et al. [23] in the Seattle Diabetic Foot Study found the mean duration of diabetes to be 13.2 years compared to 6.4 years in this study. Furthermore, some patients (12.3%) in our study have undiagnosed diabetes when they presented with foot complications. With regards to diabetes control, 81% of patients had poor glycemic control, i.e. HbA1c > 8%. For a variety of reasons, good glucose control is not easily obtained in many Indonesian patients [24]; poor drug compliance, lack of financial resources, and poor access to medical facilities may all compound this problem. Overall mean HbA1c in this study was 11.2%, higher than what Hartemann-Heutier et al. [25] and Ozkara et al. [26] have shown (mean HbA1c 8.7% and 10.3%, respectively). Thewjitcharoen et al. [27] found that approximately 56.8% of DFU patients had neuropathy only and another 29.3% had neuroischemic ulcers (42.9% and 29.9%, respectively, in our study). Of note, pure ischemic ulcers usually present in lower percentage (10.7% in our study).

A quarter of our patients were unaware of the cause of the ulcers. Perhaps the co-existence of neuropathy, lack of foot care is the main cause of the tendency for progression of their lesions before presentation. Other studies quote inadequate footwear [14,16] or spontaneous blisters [27,28] as the most common cause of foot ulceration. It is known that a previous DFU history increases the risk of further lesions [14], and carries a similar risk of LEA to those for first ulceration [29]. Recurrence of foot ulceration in 35.4% of patients showed that they require more personal education regarding foot care. This finding is very important as foot ulceration is a preventable entity in many cases with adequate education, routine foot care and attention to foot wear [19,30].

Another component to ulcer recurrences include prior partial foot amputation [30]. Even partial amputation of the great toe contributes to the development of foot deformities that increased the risk of re-ulceration [31]. The authors found that the most common level of LEA in this study was at the level of the toes (67.2%). High prices of customized footwear and the unavailability of offloading devices may provide an important factor for the high recurrence rate of DFU in our studied population. New techniques of tendon balancing have been shown to speed healing of ulcers, decrease ulcer recurrence and LEA. This approach could be incorporated as part of initial treatment of foot ulcers in the future [32].

The rate of LEA in our study was 36.3%, excluding those who refused amputation and discharged home. Although not all Indonesian studies [6,7] have been designed to evaluate LEA rates, the rates from these various studies range from 23 to 39.5%, that seems similar to our finding. In the Netherlands [33] and England [34], the amputation rates were found to be lower at 15.5% and 16.0%, respectively, related to better preventative measures and standard of care in Europe. As can be seen from Table 2, patients classified as Wagner grade 3, 4, and 5 disease accounted for a total 71.5% of cases. This reflects that most of our patients had late presentation with deep ulcer, osteomyelitis, and frank gangrene of the foot. Although individual surgeon practice may affect the decision to amputation, many studies have reported the association between higher Wagner grade with more extensive surgical intervention [9,35]. The mortality was also relatively high in this study (10.7%). The reason can be explained by the fact that some of the patients were admitted with advanced DFU and sepsis, leading to multiple organ failure and death. The actual figure of LEA incidence and mortality rate in this study could be higher in those who refused surgery or self-discharged from hospital.

It was observed that the mean duration of hospital stay for diabetic foot problem was 17.8 days, comparable with Ozkara et al.’s [26] report of an average of 17.2 days. In studies from England [36], Tanzania [37], and Nigeria [28], the mean duration of hospital stay was 22.2, 36.2 days, and 60.3 days, respectively. The variation from study to study might be related to differences in clinical practice, severity of illness, and availability of supportive care in their hospital. However, the relatively lower duration of hospitalization in the present study may be a result of death at early date or discharge from the hospital. Meanwhile, our rate is longer than the reported rate for patients who routinely examined and attend outpatient diabetic foot service (mean length of stay of only 7.1 days) when hospitalization is required [38]. This is a persuasive argument for the provision of diabetic foot care at a very early stage to reduce both the necessity and length of hospital admission and also improve patient’s outcome.

Our previous study [9] reported that the presence of PAD was found to be a major predictive factor for poor outcome after DFU admission. In low income countries, scarce resources may limit the best care, as we know that most hospitals are not even equipped for vascular intervention. But even in the technologically advanced center as Dr. Kariadi General Hospital, management is often lacking or delayed. Distal revascularization procedures were not performed in some of our patients because they have not been considered suitable by the vascular surgeon. The reason varies from severely ill patients, short life expectancy, severe nephropathy, and extensive tissue destruction. Indeed there are patients who are suitable but resist treatment because of financial issue or just simply fear of operating room. The famous maxim told that prevention is better than cure can be applicable: identification of high risk foot with simple equipment and management at early stage of ulcer is still the most efficient and cost-effective solution, rather than managing them in the hospital.

Finally, in this tertiary care hospital-based retrospective study, LEA incidence and mortality could be less related to insufficient resources than to patient’s attitudes and systems of health-care organization. Diabetic foot complications are indeed one of the preventable and curable complications of diabetes [19,39], and the current scenario should encourage us to work harder in this field. Our ultimate target should be to make effective preventative foot-care available and education programmed that will work effectively, especially in primary and secondary health care setting. We advocate for a regular awareness campaign at mass level to highlight the importance of good glycemic control, regular foot screening, and encouraging diabetic patients with a foot ulcer to present early to professional health care. There is also a need to ensure that medication used for diabetes should be made affordable and treatment for diabetic foot is covered by the national health insurance scheme. At the level of tertiary hospital care, a corollary to this preventative action is the development of teamwork and establishing a diabetic foot clinic with well-trained staff [25,27].

## Future research

There is paucity of information on the reason for high DFU prevalence and predictive values of risk factors for DFU in the Indonesian health care and community setting. Further research should also conduct studies in order to investigate the patients’ attitude with respect to knowledge and practice of foot care in Central Java province. In-depth information gained from current reports and future study will be useful in developing risk-assessment model for a larger prospective cohort study. For comparison, future studies should also evaluate clinical profiles and outcomes after the development of a specialized diabetic foot center, which is our short term goal.

## Limitations of the study

Several limitations exist to the present study, just as there are limitations in retrospective studies using a hospital database and medical record review [13]. First, a selection bias may exist because physicians or hospital facilities influence the treatment modality. Second, since our study was embedded in daily clinical practice, limitations had to be set with regard to the number and type of data collected, such as size of ulcers and foot deformity for instance. We also present several variables with missing data, as presented in the tables in the text. Third, because our aims were descriptive, we did not undertake inferential statistical analysis, so we were not able to associate the clinical profile with outcome of diabetic foot problem. Lastly, this study is not population-based and represents patients referred to a tertiary care hospital. It has the advantage of characterization for the severity of foot ulceration and LEA rate. However, the samples of this study were also relative small and single center, so it clearly indicates the need for multicenter study to provide the real size of this problem.

## Conclusion

Diabetic foot pathologies are common in diabetics and pose serious health problems for developing countries. They seem to affect both sexes equally. The present study highlights the significance of patients with DFU in tertiary care hospital in the Indonesian context where diabetes is poorly controlled, there was also little awareness for foot care and delay in seeking treatment, as this will worsens the extent of tissue destruction. Our center is a tertiary referral center in which patients referred have rather advanced diseases. Many patients fail to receive timely and optimal care once present in the hospital. In the end, LEA is a common outcome of DFU who are admitted to our hospital, as well as being a notable cause of death.
